# Supplementary material for: Integrating gene expression data into a genome-scale metabolic model to identify reprogramming during adaptive evolution
Source: PLoS One. 2023 Oct 3;18(10):e0292433. doi: 10.1371/journal.pone.0292433 (PMC10547208; doi:10.1371/journal.pone.0292433)
Supplement: S1 File — (DOCX) [file pone.0292433.s001.docx]

Integrating gene expression data into a genome-scale metabolic model to identify reprogramming during adaptive evolution


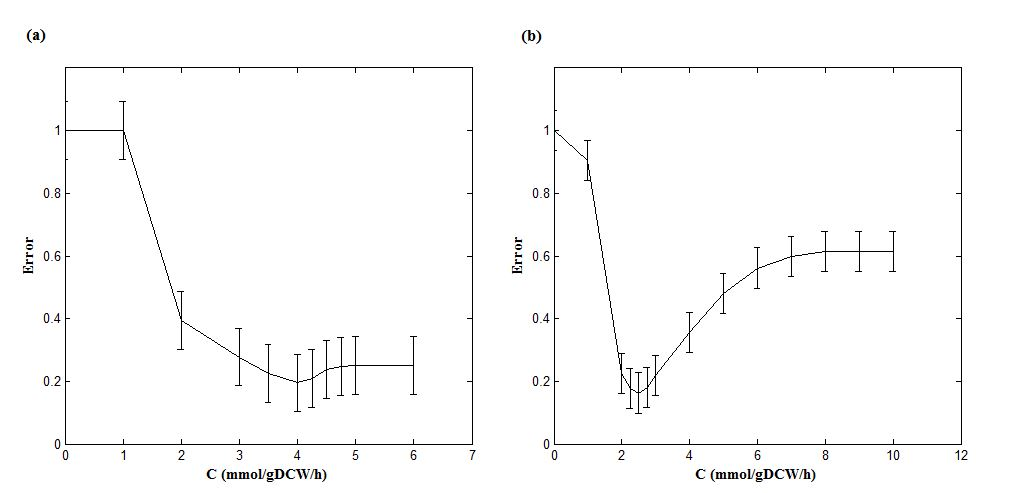
Shaghayegh Yazdanpanah, Ehsan Motamedian and Seyed Abbas Shojaosadati

**Fig S1.** Sensitivity analysis of TRFBA to parameter C in culture with (a) lactate and (b) glycerol as carbon source

**Table S1.** Adaptation score under condition of maximum flux of lactate transport reaction (lactate adaptation score) and under maximum energy production through ATP synthase (ATP adaptation score)

| Strain | Lac2 | Lac3 | LacA | LacB | LacC | LacD | LacE | WT |
| --- | --- | --- | --- | --- | --- | --- | --- | --- |
| L-lactate adaptation score | 34 | 35 | 37 | 34 | 36 | 35 | 37 | 35 |
| ATP adaptation score | 64 | 37 | 53 | 33 | 58 | 49 | 41 | 51 |


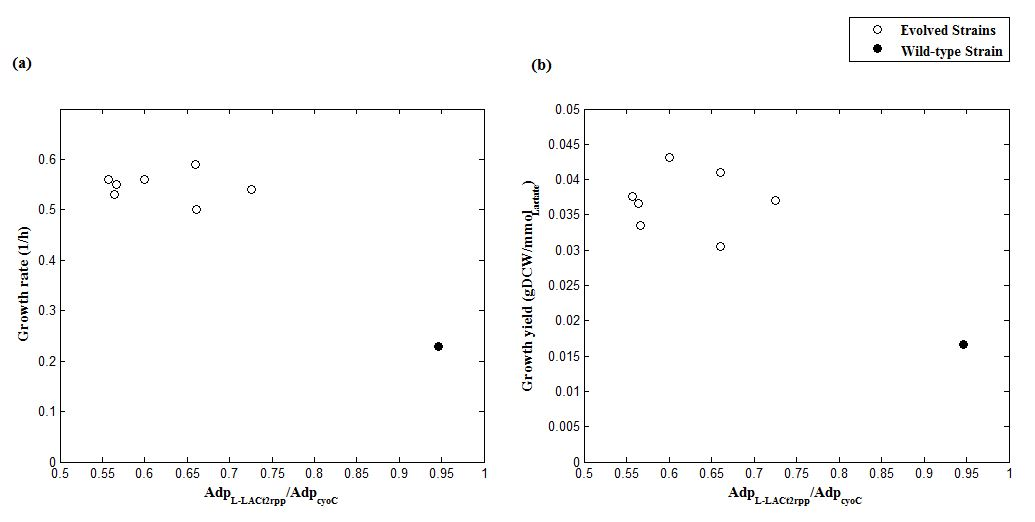


**Fig 2.** Correlations between experimental phenotypic data [1] and lactate to *cyoC* adaptation score ratio. Plots of (a) growth rate and (b) growth yield versus the ratio of lactate to *cyoC* adaptation score. *cyoC* adaptation score was calculated under the condition of maximum use of *cyoC* gene. Lactate adaptation score was calculated under the maximum flux of reaction L-LACt2rpp which transfer lactate through inner membrane. Open circles denote evolved strains at the endpoint of adaptive evolution and solid circles represent wild-type strain.

**Table S2.** Pearson coefficient between ratio of lactate to *cyoC* adaptation score and experimental data [1] obtained from culture with lactate as carbon source

|  | Growth rate (1/h) | Growth yield (gDCW/mmol_Lactate_) |
| --- | --- | --- |
| Pearson coefficient | -0.87 | -0.80 |
| p-value | 0.0041 | 0.0153 |

**
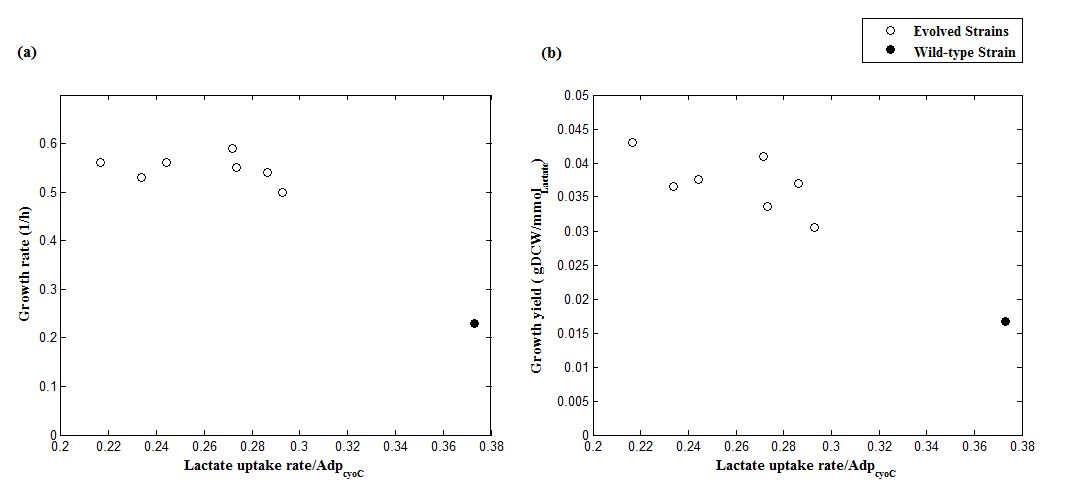
**

**Fig S3.** Correlations between experimental phenotypic data [1] and ratio of lactate uptake rate to *cyoC* adaptation score. Plots of (a) growth rate and (b) growth yield versus experimental lactate uptake rate to *cyoC* adaptation score ratio. *cyoC* adaptation score was calculated under the condition of maximum use of *cyoC* gene. Open circles denote evolved strains at the endpoint of adaptive evolution and solid circles represent wild-type strain.

**Table S3.** Pearson coefficient between ratio of experimental lactate uptake rate [1] to *cyoC* adaptation score and experimental data obtained from culture with lactate as carbon source

|  | Growth rate (1/h) | Growth yield (gDCW/mmol_Lactate_) |
| --- | --- | --- |
| Pearson coefficient | -0.90 | -0.85 |
| p-value | 0.0020 | 0.0073 |


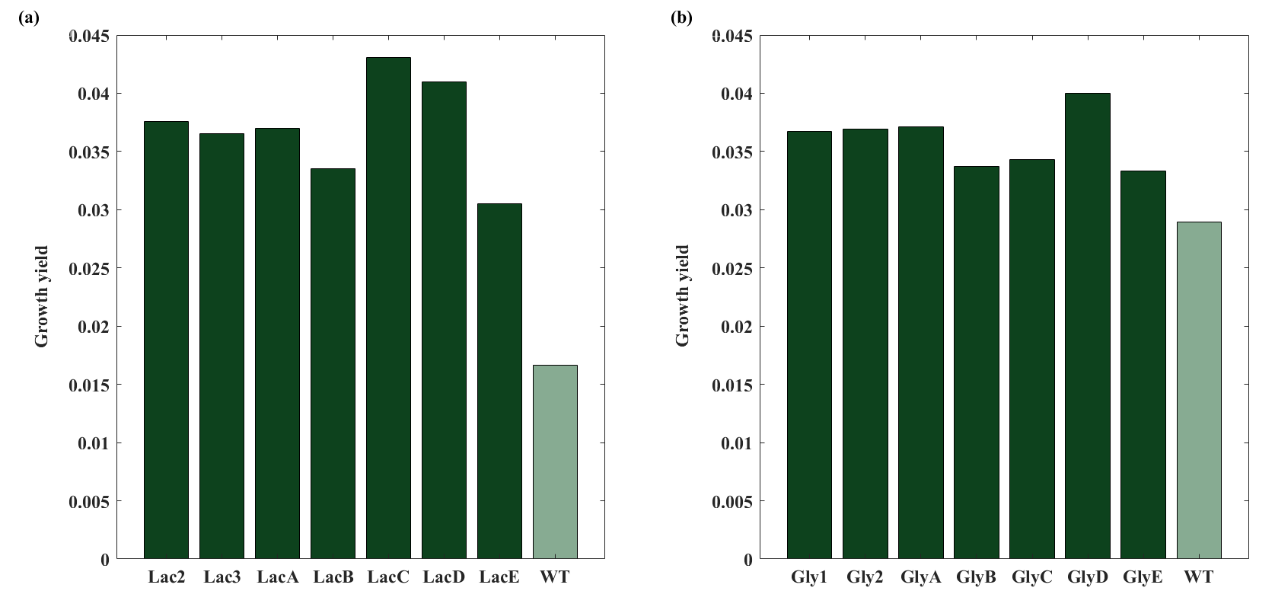


**Fig S4.** Experimental growth yield of wild-type and evolved strains in culture medium containing (a) lactate and (b) glycerol as carbon source [1]

References

1. Fong SS, Joyce AR, Palsson BØ. Parallel adaptive evolution cultures of *Escherichia coli* lead to convergent growth phenotypes with different gene expression states. Genome Res. 2005; 15: 1365-1372.
